# Effects of brain-computer interface training on upper limb function recovery in stroke patients: A protocol for systematic review and meta-analysis

**DOI:** 10.1097/MD.0000000000026254

**Published:** 2021-06-11

**Authors:** Xiali Xue, Huan Tu, Zhongyi Deng, Ling Zhou, Ning Li, Xiaokun Wang

**Affiliations:** a Institute of Sports Medicine and Health, Chengdu Sport University; b School of Sports Medicine and Health, Chengdu Sport University, Chengdu, Sichuan Province; c The People's Hospital of Mancheng District, Baoding, Hebei Province, China.

**Keywords:** brain-computer interface, meta-analysis, protocol, stroke, upper limb function

## Abstract

**Background:**

In recent years, with the development of medical technology and the increase of inter-disciplinary cooperation technology, new methods in the field of artificial intelligence medicine emerge in an endless stream. Brain-computer interface (BCI), as a frontier technology of multidisciplinary integration, has been widely used in various fields. Studies have shown that BCI-assisted training can improve upper limb function in stroke patients, but its effect is still controversial and lacks evidence-based evidence, which requires further exploration and confirmation. Therefore, the main purpose of this paper is to systematically evaluate the efficacy of different BCI-assisted training on upper limb function recovery in stroke patients, to provide a reference for the application of BCI-assisted technology in stroke rehabilitation.

**Methods:**

We will search PubMed, Web of Science, The Cochrane Library, Chinese National Knowledge Infrastructure Database, Wanfang Data, Weipu Electronics, and other databases (from the establishment to February 2021) for full text in Chinese and English. Randomized controlled trials were collected to examine the effect of BCI-assisted training on upper limb functional recovery in stroke patients. We will consider inclusion, select high-quality articles for data extraction and analysis, and summarize the intervention effect of BCI-assisted training on the upper limb function of stroke patients. Two reviewers will screen titles, abstracts, and full texts independently according to inclusion criteria; Data extraction and risk of bias assessment were performed in the included studies. We will use a hierarchy of recommended assessment, development, and assessment methods to assess the overall certainty of the evidence and report findings accordingly. Endnote X8 will be applied in selecting the study, Review Manager 5.3 will be applied in analyzing and synthesizing.

**Results:**

The results will provide evidence for judging whether BCI is effective and safe in improving upper limb function in patients with stroke.

**Conclusion:**

Our study will provide reliable evidence for the effect of BCI technology on the improvement of upper limb function in stroke patients.

**PROSPERO registration number:**

CRD42021250378.

## 1 Introduction

Stroke is a global problem, affecting all races, genders, and ages.^[1]^ According to statistics, about 5.5 million people died from stroke every year worldwide, and 5 million people suffered from permanent disability due to stroke.^[2]^ Stroke has become the second leading cause of death and stroke, and it is also the main cause of long-term severe disability.^[3,4]^ About 30% of stroke survivors have severe dyskinesia, and they need the help of daily life activities.^[5]^ A large number of studies have shown that the rehabilitation difficulty of the upper limb is higher than that of the lower extremity,^[6–9]^ and about 70% of patients have left upper limb dysfunction, which leads to a serious decline in the quality of life.^[10]^ Despite tremendous efforts over the past few decades, people have never stopped seeking better treatments to help improve upper limb function in stroke patients.^[11]^

A brain-computer interface (BCI) is a system that allows users to control devices in the environment through neural activity.^[12]^ It only allows the control of computers through brain activity without muscle control, providing direct communication pathways between the human brain and external devices.^[13]^ There are 2 main categories of brain-computer interfaces: implantable and noninvasive, which are distinguished by invasive and noninvasive brain signals. Brain-computer interface technology is an exciting advance in neuroscience and engineering. In 1968, the world's first BCI technology had been applied to the occipital cortex of blind people, and patients could see a flash when stimulated.^[14]^ With the development of science and technology, BCI as a new, frontier, painless intervention has been gradually applied to stroke rehabilitation, so that patients participate in BCI feedback training.^[15–18]^ In the motor-computer interface, electronic records from the motor cortex of the paralyzed person are decoded by the computer, converting brain signals into commands to control external equipment, used to drive the manipulator or to restore the movement of the paralyzed hand by stimulating the muscles of the forearm. It is considered to be a powerful tool for the rehabilitation of stroke patients.^[19–21]^

At present, there is a lot of published and advanced knowledge about BCI in stroke, and it is necessary to update the evidence continuously and conduct more in-depth analysis. Therefore, the main purpose of this study was to evaluate the effectiveness and systematic scientific basis of brain-computer interface intervention in upper limb functional rehabilitation of stroke patients. In addition, this paper plans to propose some suggestions for future research in this field through the results of this study.

## 2 Methods

### 2.1 Study registration

The protocol of our study has been registered with the International Prospective Register of Systematic Reviews (PROSPERO) (registration number: CRD42021250378). The protocol is reported strictly according to the Preferred Reporting Items for Meta-Analyses Protocols (PRISMA-P) guidelines.

### 2.2 Eligibility criteria

#### 2.2.1 Type of study

We will include the randomized controlled trials of brain-computer interface-assisted training to improve upper limb function in stroke patients.

#### 2.2.2 Type of participant

Stroke patients aged 18 to 80 years (following the clinical diagnostic criteria for stroke) need to be diagnosed by cranial CT or MRI. The course of the disease was subacute (onset 1–3 months) or chronic (onset >3 months) stroke accompanied by upper limb dysfunction without severe cognitive impairment. Compared with the general data of sex, stroke type, hemiplegia, age, and course of the disease, there was no significant difference between the 2 groups (*P* > .05). Its race, nationality, sex, unlimited.

#### 2.2.3 Type of intervention

Our research will include studies in which the intervention measures for the experimental group were to receive conventional rehabilitation training and brain-computer interface training, while the control group received conventional rehabilitation therapy (physical therapy, occupational therapy, etc.), sham therapy, or other exercise programs.

#### 2.2.4 Types of outcome measurements

The primary outcome will be used to assess the improvement of upper limb function in stroke patients, and secondary results will be used to assess the ability to perform activities of daily living. The main evaluation index was the upper limb motor function score (Fugl-Meyer Upper Limb Motor Function, FMA-UE), and the higher the score, the better the upper limb function. And other relevant indicators for reference.

#### 2.2.5 Inclusion criteria

We will use the PICOS (Participants, Intervention, Comparator, Outcome, and Study design) model to select studies for this review. The inclusion criteria were as follows:

1. Participants: patients with stroke;
2. Intervention: patients received brain-computer interface;
3. Comparator: patients received other treatment;
4. Outcomes: the primary outcome will be used to assess the improvement of upper limb function, and secondary results will be used to assess the ability to perform activities of daily living;
5. Study design: Randomized clinical trial.

#### 2.2.6 Exclusion criteria

1. Non-Chinese and English literature;
2. Participants without clear diagnosis;
3. Outcome index data are missing;
4. Patients before or after the group of multiple therapies rehabilitation treatment.
5. Case reports, repeated publication, too little information, incomplete data and only abstract but no full text, unable to use the literature.

### 2.3 Search methods for identification of studies

#### 2.3.1 Electronic data sources

The following electronic databases will be searched from inception to February 2021: PubMed, the Cochrane Library, Web of Science, China National Knowledge Infrastructure, WanFang Data, Weipu Electronics. In addition, reference lists of the included studies were manually searched to identify additional relevant studies.

#### 2.3.2 Other resources

Relevant references will be reviewed and screened. In addition, we will search the following registration website of the clinical trial: WHO ICTRP, http:// www.chictr.org.cn, http://www.ClinicalTrial.gov, and ISRCTN Register. Moreover, the relevant grey literature from the Health Management Information Database (HMIC), Open SIGLE Database, and the National Technical Information Service (NTIS) will be searched. Experts in the field will be consulted for relevant studies.

### 2.4 Search strategy

The search is performed by combining subject terms with free terms. The search terms on PubMed are Brain-computer interface (e.g., Brain computer interface or Brain machine interface); Stroke (e.g., stroke or cerebrovascular accident or stroke); Randomized controlled trials (e.g., randomized or randomized or clinical trials). Combinations of Medical Subject Headings (MeSH) and text words will be used. The same search terms are used in other electronic databases. These search terms are shown in Table 1. Different databases have different characteristics and different retrieval strategies.

**Table 1:** Search strategy for the PubMed database.

Number	Search items
1	Brain Computer Interface
2	Brain Computer Interfaces
3	Interface, Brain-Computer
4	Interfaces, Brain-Computer
5	Brain-Computer Interface
6	Brain Machine Interface
7	Brain-Machine Interfaces
8	Brain Machine Interfaces
9	Brain-Machine Interface
10	Interface, OR Brain-Machine
11	Interfaces, Brain-Machine
12	1 or 10–11
13	Stroke
14	Strokes
15	Cerebrovascular Accident
16	Cerebrovascular Accidents
17	CVA (Cerebrovascular Accident)
18	CVAs (Cerebrovascular Accident)
19	Cerebrovascular Apoplexy
20	Apoplexy, Cerebrovascular
21	Vascular Accident, Brain
22	Brain Vascular Accident
23	Brain Vascular Accidents
24	Vascular Accidents, Brain
25	Cerebrovascular Stroke
26	Cerebrovascular Strokes
27	Stroke, Cerebrovascular
28	Strokes, Cerebrovascular
29	Apoplexy
30	Cerebral Stroke
31	Cerebral Strokes
31	Stroke, Cerebral
33	Strokes, Cerebral
34	Stroke, Acute
35	Acute Stroke
36	Acute Strokes
37	Strokes, Acute
38	Cerebrovascular Accident, Acute
39	Acute Cerebrovascular Accident
40	Acute Cerebrovascular Accidents
41	Cerebrovascular Accidents, Acute
42	13 or 14–41
43	Randomized controlled trial
44	Randomized
45	Clinical trial
46	Randomly
47	Controlled clinical trials
48	Controlled before-after studies
49	43 or 44–48
50	12 and 42 and 49

### 2.5 Data collection

#### 2.5.1 Selection of studies

**Figure 1:**
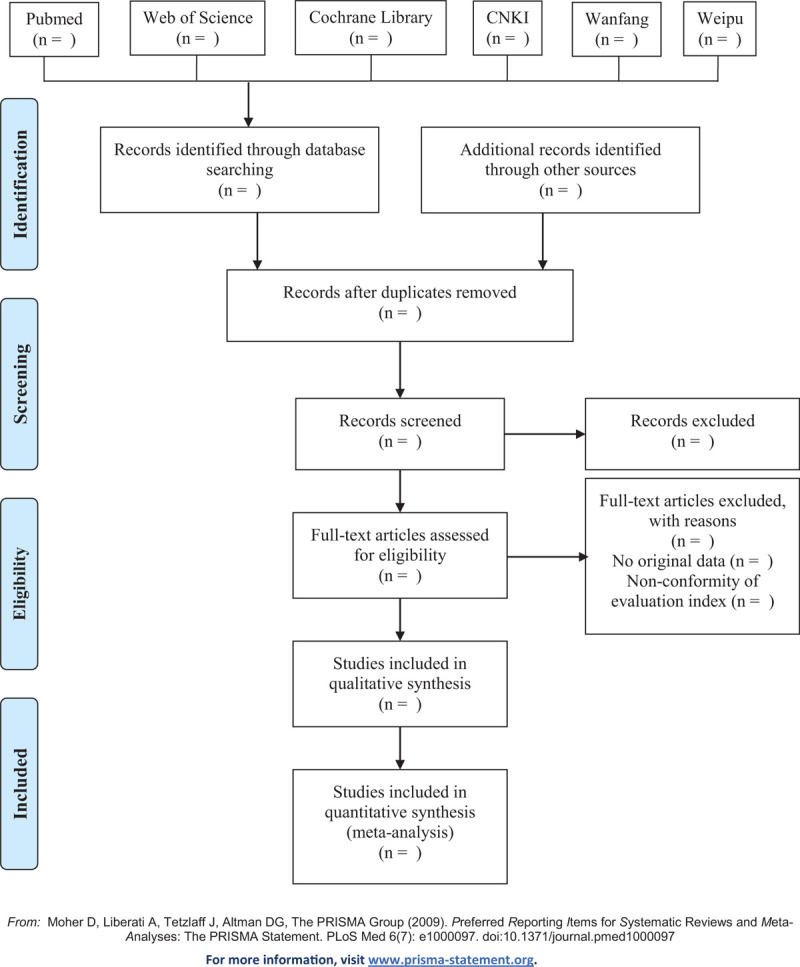
Flow diagram of the study selection process.

The retrieved studies will be imported in Endnote X8 to remove duplicates. Two researchers (XLX and HT) will screen the titles and abstracts independently according to the pre-established inclusion and exclusion criteria. After that, the full text will be screened as a second filtration. Two researchers will crosscheck the included studies, and the third researcher (NL) will be involved if disagreements occur. The detailed screening process will be shown in the following Preferred Reporting Items for Systematic Reviews and Meta-Analyses Protocols flow diagram (Fig. 1).

#### 2.5.2 Data extraction and management

The other 2 researchers (LZ and ZYD) will extract data independently to fill out the predesigned form. The information includes author, country, publication year, methodological quality, characteristics of participants, the details of intervention and comparisons, outcomes, the specific data, results, conclusions, follow-up, adverse events, conflicts of interest, sources of funds, and ethical approval. The extracted data will be crosschecked by the 2 researchers. A third researcher (NL) will be involved in a disagreement occurs. The authors of the studies included will be contacted for further information when necessary.

#### 2.5.3 Risk assessment of bias included in the study

Two researchers (XLX and ZYD) independently evaluated the risk of bias included in the study, and finally the summary. In the absence of consensus, evaluated by the third person. The bias risk assessment was by a randomized controlled trial bias risk assessment tool recommended by the international Cochrane manual 5.1.0.^[22]^ The main contents of literature quality assessment include:

1. Whether to group randomly;
2. Whether concealment is assigned;
3. Blindness (participants, implementers, evaluators);
4. Integrity of the resulting data;
5. Selective reporting;
6. Other sources of bias.

The risk of low bias is expressed as yes, the risk of high bias is expressed as no, and the information not mentioned in the article is expressed as unclear.

### 2.6 Data synthesis

RevMan5.3 software is used to analyze data. Effect calculation: a study using the same results, the mean difference, and the corresponding 95% confidence interval. If you use different results, and the standardized mean difference of the mean change measured by the selected results is weighted standard deviation mean difference standardized mean difference.$x^{2}$ the test is used to test whether the combined statistics of multiple similar studies have significant significance, and the probability *P* value of the statistic is worth according to the$x^{2}$. If *P* < .05, then the combined statistics of multiple studies have significant significance; If *P* > .05, the combined statistics of multiple studies are not significant. Heterogeneity test: the heterogeneity of intervention effect is inevitable, because of the differences in the design of the study. The heterogeneity among the results was analyzed by $x^{2}$ test (*P* = .10), at the same time, combined with *I*^2^ quantitative judgment of heterogeneity. If *P* > .10, *I*^2^ < 50%, which indicates that the studies are homogeneous, fixed effect model analysis should be selected; If *P* ≤ .10, *I*^2^ > 50%, which indicates heterogeneity among the studies, a random effect model should be selected for analysis. The level of Meta-analysis is set to *P* < .05. For obvious heterogeneity, subgroup analysis or sensitivity analysis were used, or only descriptive analysis.

#### 2.6.1 Management of missing data

The related corresponding author will be contacted if there are insufficient or missing data. If accurate data is still unavailable after contacting the corresponding author, these studies will be excluded.

#### 2.6.2 Assessment of reporting biases

If the quantity of the included randomized controlled trials was no less than 10, funnel plots will be selected to evaluate the potential publication bias.

#### 2.6.3 Subgroup analysis

Subgroup analysis was carried out according to different BCI treatment modes, different intervention modes in the control group, different time points of patients’ disease course, intervention cycle, and posttreatment evaluation results.

#### 2.6.4 Sensitivity analysis

Based on the risk of bias, insufficient data, and sample size, we will perform a sensitivity analysis to evaluate the robustness of significant statistical heterogeneity existed.

### 2.7 Grading the quality of evidence

This paper will use the evidence quality rating method to evaluate the results obtained from this analysis. GRADE will be assessed across the domains of risk of bias, consistency, directness, precision, and publication bias. In the context of the system review, quality reflects our confidence in the effectiveness of the assessment. It has 4 evaluation levels, namely, high (further research is very unlikely to change our confidence in the estimate of effect), moderate (further research is likely to have an important impact on our confidence in the estimate of effect and may change the estimate), low (further research is very likely to have an important impact on our confidence in the estimate of effect and is likely to change the estimate), or very low (very uncertain about the estimate of effect).

### 2.8 Ethics and dissemination

The present study will use published data and does not require ethics approval.

## 3 Discussion

Stroke is also the main cause of death of cerebrovascular diseases in China, accounting for about one-third of the global stroke mortality rate.^[23]^ In recent years, the average age of stroke patients in China showed a downward trend, and the average onset age was about 63 years old.^[24]^ As a result, middle-aged people should also pay attention to prevention more than treatment, reduce the risk factors that can be changed, hypertension, diabetes, smoking, and lifestyle factors, such as obesity, diet, malnutrition, and lack of physical activity.^[25]^ Stroke often leads to a variety of motor, sensory, cognitive, and other injuries, and it is also the main cause of distal upper limb dysfunction, which is caused by nerve injury. Hence, successful rehabilitation therapy must promote neuronal connections remaining in the brain.^[26]^

As a new and cutting-edge technology, BCI can assist stroke patients in upper limb rehabilitation training with high acceptability and less pain. BCI provides a gateway to brain plasticity and changes the way humans interact with the world. Determining whether brain-computer interface technology is a good option for stroke patients is crucial. Studies have shown that BCI-assisted training can effectively improve the upper limb function of stroke patients, but its efficacy has not been evaluated scientifically and systematically. The purpose of this study was to evaluate the efficacy and safety of BCI-assisted training for the upper limbs of stroke patients, with the hope that this review could provide more evidence. This review has some limitations. For example, different types of BCI technology models and different routine rehabilitation training methods may have heterogeneous risks. In addition, the measurements and tools that will be used to include the results of the studies may differ.

However, there might be some potential limitations in this study: low quality of original researches, different dosages, and frequency of intervention, various duration of disease, language restriction, and so on.

## Author contributions

**Conceptualization:** Xiali Xue.

**Data curation:** Zhongyi Deng, Ling Zhou.

**Formal analysis:** Xiali Xue, Huan Tu.

**Funding acquisition**: Ning Li.

**Investigation:** Zhongyi Deng, Xiaokun Wang.

**Methodology:** Xiali Xue, Huan Tu, Ling Zhou.

**Software:** Xiali Xue, Huan Tu.

**Writing – original draft:** Xiali Xue.

**Writing – review & editing:** Xiali Xue, Huan Tu, Ning Li.
